# Immune Checkpoint Inhibitor-Induced Myocarditis vs. COVID-19 Vaccine-Induced Myocarditis—Same or Different?

**DOI:** 10.3390/life12091366

**Published:** 2022-09-01

**Authors:** Lior Zornitzki, Ofer Havakuk, Zach Rozenbaum, Dana Viskin, Yaron Arbel, Nir Flint, Joshua Arnold, Barliz Waissengein, Ido Wolf, Shmuel Banai, Yan Topilsky, Michal Laufer-Perl

**Affiliations:** 1 Department of Internal Medicine B, Tel Aviv Sourasky Medical Center, 6 Weizman Street, Tel Aviv 6423906, Israel; 2 Sackler School of Medicine, Tel Aviv University, Tel Aviv 6423906, Israel; 3 Department of Cardiology, Tel Aviv Sourasky Medical Center, 6 Weizman Street, Tel Aviv 6423906, Israel; 4 Department of Cardiology, Tulane University, New Orleans, LA 70112, USA; 5 Department of Medicine, University of Illinois at Chicago, Chicago, IL 60612, USA; 6 Devision of Oncology, Tel-Aviv Sourasky Medical Center, Tel Aviv 6423906, Israel

**Keywords:** ICIs, COVID-19, vaccine, myocarditis, speckle strain, cardio-oncology

## Abstract

Immune checkpoint inhibitor (ICI) and coronavirus disease 2019 (COVID-19) vaccine-induced myocarditis possibly share common mechanisms secondary to overactivation of the immune system. We aimed to compare the presenting characteristics of ICIs and COVID-19 vaccine-induced myocarditis. We performed a retrospective analysis of characteristics of patients diagnosed with either ICIs or COVID-19 vaccine-induced myocarditis and compared the results to a control group of patients diagnosed with acute viral myocarditis. Eighteen patients diagnosed with ICIs (ICI group) or COVID-19 vaccine (COVID-19 vaccine group)-induced myocarditis, and 20 patients with acute viral myocarditis (Viral group) were included. The ICI group presented mainly with dyspnea vs. chest pain and fever among the COVID-19 vaccine and Viral groups. Peak median high sensitivity Troponin I was markedly lower in the ICI group (median 619 vs. 15,527 and 7388 ng/L, *p* = 0.004). While the median left ventricular (LV) ejection fraction was 60% among all groups, the ICI group had a lower absolute mean LV global longitudinal strain (13%) and left atrial conduit strain (17%), compared to the COVID-19 vaccine (17% and 30%) and Viral groups (18% and 37%), *p* = 0.016 and *p* = 0.001, respectively. Despite a probable similar mechanism, ICI-induced myocarditis’s presenting characteristics differed from COVID-19 vaccine-induced myocarditis.

## 1. Introduction

Immune checkpoint inhibitors (ICIs) and coronavirus disease 2019 (COVID-19) vaccine-induced myocarditis are rare, but significant manifestations, capable of causing substantial morbidity. Both complications are thought to be triggered by the overactivation of the host’s immune system cascading to a targeted attack against the myocardium [1,2].

The mechanism of inadvertent myocardial injury from ICI therapy administration is likely T-cell receptor-mediated and occurs through direct targeting of a shared antigen on myocardial tissue and tumor, and/or through the targeting of programmed cell death-ligand1 (PD-L1) in the myocardium [3]. ICI-induced myocarditis has a variable presentation and can range from mild to fulminant symptomatology. While most patients present with elevated serum troponin levels, the prevalence of reduced left-ventricle ejection fraction (LVEF), right ventricular dysfunction, and clinical heart failure has been found to be less common [4,5]. Left ventricle global longitudinal strain (LVGLS) measurement using two-dimensional speckle tracking echocardiography (2D–STE) is a widely used tool for the detection and diagnosis of early subclinical myocardial injury and is the gold standard for the assessment and follow-up in cancer patients treated with cardiotoxic regimens [6]; however, data are still limited in the setting of ICI-induced myocarditis. COVID-19 vaccine-induced myocarditis is recognized as a rare complication of the anti-COVID-19 mRNA vaccines. Although the exact mechanism of this phenomenon has not yet been fully elucidated, possible mechanisms include recognition of vaccine mRNA as an antigen by the host’s immune system, leading to an immunologic cascade of autoantibody development and an increase in Natural Killer cell quantity and activity [7]. COVID-19 vaccine-induced myocarditis typically occurs in young men and most often after the second dose of the vaccination. Patients commonly present with elevated serum troponin levels and preserved LVEF [8], and the course of COVID-19 vaccine-induced myocarditis seems to be mild and most patients recover fully [9,10]. Nevertheless, data regarding the characteristics of COVID-19 vaccine-induced myocarditis and 2D–STE evaluation is still lacking.

Based on the proposed common mechanistic pathways of stimulating the immune system, we aimed to evaluate and describe the clinical, laboratory, and imaging characteristics of patients diagnosed with ICIs and COVID-19 vaccine-induced myocarditis. We assess whether they possess different presenting characteristics and outcomes compared to the “classical” viral myocarditis, which is considered to develop in a different mechanism, in the presence of acute infection.

## 2. Materials and Methods

### 2.1. Study Design and Population

We performed a retrospective, single-center, observational study at the Tel-Aviv Sourasky Medical Center, a large tertiary hospital. The cohort included 3 groups: the ICI group included all patients evaluated at the cardio-oncology clinic and diagnosed with ICI-induced myocarditis according to recent literature [4]. The COVID-19 vaccine group included all patients hospitalized and diagnosed with COVID-19 vaccine induced-myocarditis, after receiving the second dose of the BioNTech BNT162b2 COVID-19 (Pfizer) vaccine. The Viral group was a control group and included patients hospitalized and discharged with the diagnosis of acute viral myocarditis, according to the treating physician. The study was approved by the Tel-Aviv Sourasky medical center Institutional Review Board. 

### 2.2. Definition of Myocarditis 

The diagnosis of myocarditis was made according to the European Society of Cardiology (ESC) guidelines of myocarditis 2013 [11]. Because none of the patients underwent an endomyocardial biopsy, the diagnosis of myocarditis was made based on the patient’s clinical presentation as assessed by cardiac symptoms defined as chest pain, dyspnea, left or right heart failure, elevated serum cardiac enzyme, including high sensitivity troponin I (hs-TnI) > 50 ng per liter (ng/L) or BNP > 100 picogram/milliliter (PG/ML), abnormal electrocardiogram (ECG), decreased LVEF (<50%) by a transthoracic echocardiogram (TTE), or abnormal Cardiac Magnetic Resonance (CMR) typical for myocarditis, according to the classical Lake Louise criteria and latter according to the updated criteria.

### 2.3. Study Protocol

All study participants completed a medical history evaluation which included a review of chronic diseases, cardiac risk factors, medical treatment, and laboratory results such as high sensitivity C-reactive protein (CRP), creatine phosphokinase (CPK), and hs-TnI. All patients underwent a comprehensive TTE assessment, including 2D–STE, as described in the echocardiography section. CMR (MAGNETOM Aera 1.5T, Siemens, Erlangen, Germany) was performed according to the decision of the treating physician. 

### 2.4. Assessment of Echocardiographic Characteristics

Routine left ventricle (LV) echocardiographic parameters included LV diameters and LVEF [12]. Early trans-mitral flow velocity (E), late atrial contraction (A) velocity, deceleration time (DT), and early diastolic mitral annular velocity (septal and lateral e’) were measured in the apical 4-chamber (4C) view to provide an estimate of LV diastolic function [13]. The peak E to peak e’ ratio (E/e’) was calculated (average mitral E/e’ ratio) [14]. Tricuspid annular plane systolic excursion (TAPSE) was measured as the distance of systolic excursion of the right ventricle (RV) annular segment along its longitudinal plane, using M-mode from the apical 4C window [12,15,16].

### 2.5. 2D-STE

A semi-automated speckle-tracking analysis was performed using commercial feature tracking software (TOMTEC arena AutoSTRAIN, TomTec Imaging Systems, Unterschleissheim, Germany), in accordance with the Consensus Document of the EACVI/ASE/Industry Task Force to Standardize RV and LV myocardial Deformation Imaging [12,17]. All 2D-STE analyses were performed offline. LVGLS was obtained as an average of 18 segments from the apical two-chamber (2C), three-chamber (3C), and 4C views. An end-diastolic frame was used to standardize LV boundaries which were then automatically tracked throughout the cardiac cycle. The investigators reviewed the border tracking and performed manual corrections when needed to optimize boundary tracking. Using a 4C RV focus view, the margins of the RV were marked. RV 4C longitudinal strain (RV4CLS) is the average of 6 segments (3 of the free wall and 3 of the septum). Peak free-wall RV longitudinal strain (FWRVLS) was calculated by averaging the strain measurements of the 3 RV free-wall segments (basal, middle, and apical). Left atrial strain (LAS) was assessed from the apical 4C view with end-diastole as the baseline and reported as LAS reservoir (LASr), LAS conduit (LASc), LAS pump (LASp), as accepted by the literature [17]. All strain values are presented as absolute values where a decrease in strain (i.e., lower absolute value) is observed when LV, LA or RV function deteriorates.

### 2.6. Statistical Analysis

Continuous variables were presented as means (±standard deviation) or as medians (interquartile range) when appropriate, depending on the distribution. Categorical variables were described as absolute numbers and percentages. Analysis of Variance (ANOVA) was used to compare continuous parameters. Chi square test was used to compare categorical variables. A two-tailed *p*-value of <0.05 was considered significant. All analyses were performed with the SPSS software (SPSS Inc., Chicago, IL, USA).

## 3. Results

### 3.1. Baseline Parameters

Overall, our cohort included 38 patients, 9 patients in the ICI group evaluated in the cardio-oncology clinic between April 2016 to April 2021, 9 patients in the COVID-19 vaccine group that were hospitalized from February to April 2021, and 20 patients in the Viral group as a control. Baseline characteristics and medical therapy are presented in Table 1. Patients in the ICI group were significantly older (median, 74 years [IQR 64–79]) compared to the COVID-19 vaccine and Viral groups (20 [19–29] and 24 [22–26] years, respectively), *p* < 0.001. In all groups, female gender representative was less common (10–22%). Baseline history of cardiac diseases was uncommon in all groups; however, hyperlipidemia and hypertension were more frequent in the ICI group. Consequently, more patients in the ICI group were treated with antihypertensive drugs (22–44% vs. 0%). In the ICI group, the most common cancer was lung adenocarcinoma (44%). The majority of the ICI group were treated with monotherapy (two patients with pembrolizumab, two patients with durvalumab, two patients with nivolumab, two patients with atezolizumab), and only one patient was treated with combined therapy of nivolumab and ipilimumab. Overall, eight patients (89%) were treated with prior chemotherapy and two patients (22%) were exposed to chest radiation.

### 3.2. Clinical Presentation

Most patients in the COVID-19 vaccine and Viral group presented with chest pain (100% and 90%, respectively), and a substantial portion had a fever (78% and 40%, respectively), while these symptoms were less frequent in the ICI group (44%, *p* = 0.010 and 0%, *p* = 0.002, respectively). Patients in the ICI group presented primarily with dyspnea (67% compared to 11% and 5% in the COVID-19 vaccine and Viral groups, *p* = 0.001, respectively).

### 3.3. Laboratory Tests

Median peak hs-TnI was markedly lower in the ICI group compared to the COVID-19 vaccine and Viral groups (619 ng/L vs. 15,527 ng/L vs. 7388 ng/L, *p* = 0.004). No significant difference was observed regarding CRP levels. Median hemoglobin values were lower in the ICI group (10.6 [9.8–13.7] g/dL, vs. 15.2 [13.9–15.7] g/dL and 13.8 [13.3–14.9] g/dL, *p* = 0.001) (Table 2). No significant differences in other values were observed in the blood count.

### 3.4. ECG and Imaging Tests

ST-elevation was frequent in the COVID-19 vaccine and Viral groups and absent from the ICI group (67% and 60% versus 0%; *p* = 0.004) (Table 3).

**Figure 1:**
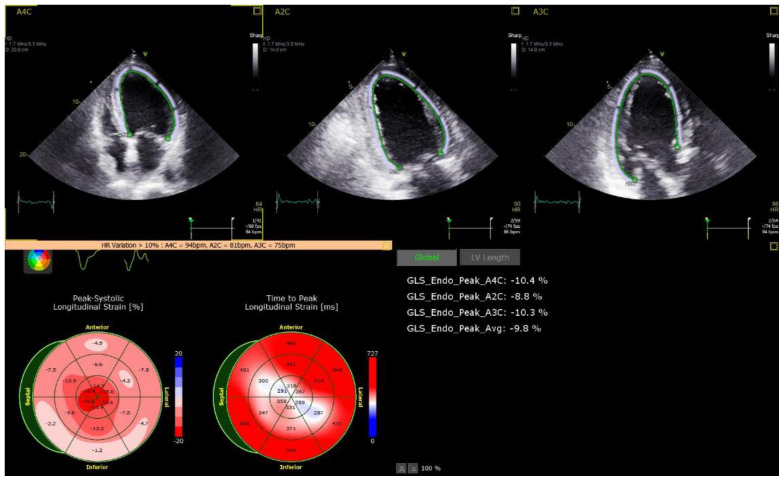
Reduced left ventricle longitudinal strain in the ICI group.

**Figure 2:**
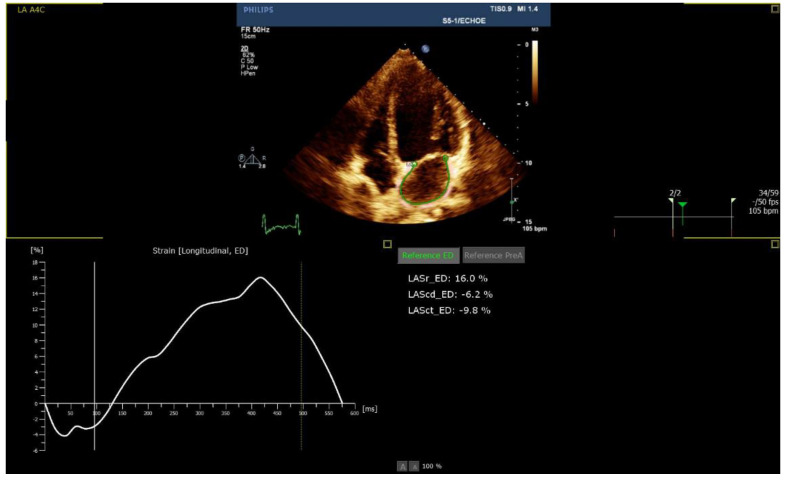
Reduced left atrium strain in the ICI group.

**Figure 3:**
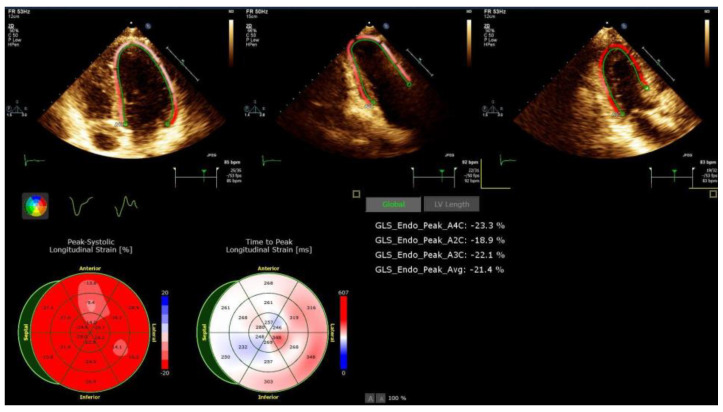
Preserved left ventricle longitudinal strain in the COVID-19 vaccine group.

**Figure 4:**
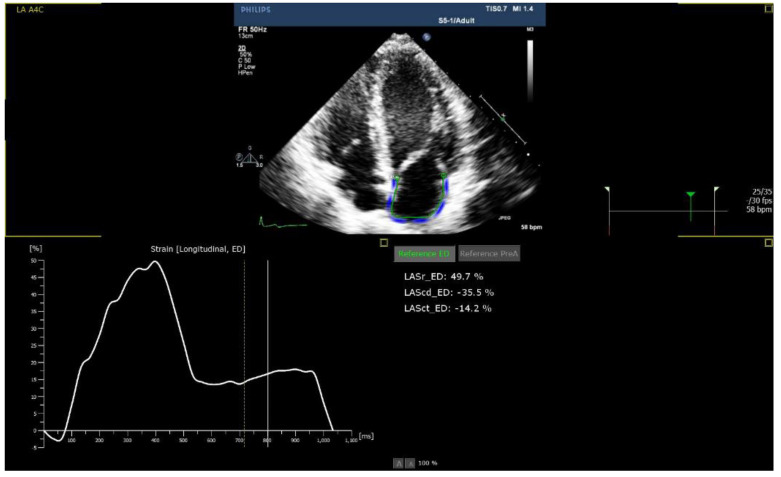
Preserved left atrium strain in the COVID-19 vaccine group.

Echocardiographic parameters are presented in Table 3. While the median LVEF was 60% among all groups, patients in the ICI group presented with lower mean absolute LVGLS (13%) (Figure 1) and LASc (17%) (Figure 2), compared to the COVID-19 vaccine (17% and 30%) and Viral groups (18%, *p* = 0.016; and 37%, *p* = 0.001, respectively; Figure 3 and Figure 4). LASr showed a trend of lower values in both the ICIs and COVID-19 vaccine groups, compared to the Viral group (32% and 33% vs. 44%, respectively, *p* = 0.066). No significant differences were observed in RV function, including TAPSE, RV4CSL, and RVFWS. Evaluation of diastolic function showed that the ICI group tended to present with diastolic dysfunction, characterized by lower e’ septal and lateral and higher E/e’ septal, lateral, and average. Five patients (56%) in the ICI group performed follow-up 2D–STE, showing improvement in LV GLS values with a mean -16.2%.

Overall, 5 (56%) patients in the ICI group and 7 (78%) patients in the COVID-19 vaccine group performed CMR. While all patients presented with late gadolinium enchantment (LGE), increased T2 and T1 was presented in 50% and 62% of the patients in the COVID-19 group and 25% and 75% of the patients in the ICI group.

### 3.5. Therapy

Significant differences were observed regarding the administration of medical therapy between groups. The ICI group received a steroid-based protocol (78%), while in both the COVID-19 vaccine and Viral groups, colchicine was administered (56% and 85%, respectively), and non-steroidal anti-inflammatory drugs (NSAIDs) were given in the Viral group (45%). A trend for more frequent use of ACEi/ARB was seen in the COVID-19 vaccine (89%) and Viral groups (60%) (Table 2).

## 4. Discussion

In our paper, we compared, for the first time to our knowledge, the presenting clinical, laboratory, and imaging features of ICIs and COVID-19 vaccine-induced myocarditis. Although the mechanism of these two diseases is suspected to be similar, we observed that the clinical manifestation of ICI-induced myocarditis was significantly different from post-COVID-19 vaccination myocarditis, with the latter being more similar to the presentation of viral myocarditis.

ICIs therapy has altered the field of cancer therapy by achieving durable antitumor responses in many advanced malignancies previously associated with poor prognoses [18]. Adverse side effects, resulting from inappropriate overactivation of the host’s immune system have been reported among patients treated with immunotherapy. ICI-induced myocarditis has been found to be rare, with an estimated prevalence of 1.14% [19] and potentially fatal adverse effect, with a mortality rate of up to 46% [20]. The suggested mechanisms include the targeting of an antigen shared on the surface of tumor and myocardial cells by ICIs leading to abnormal activation of immune pathways [1].

As a result of the COVID-19 pandemic and the extensive vaccination initiatives developed as a response, COVID-19 vaccine-induced myocarditis has emerged as a side effect of the mRNA vaccines, with an estimated incidence of 2.13 cases per 100,000 persons [2]. Although deliberate modifications to the mRNA molecules used for the vaccines have reduced their propensity to trigger innate immunogenicity, they can still cause an immunologic reaction in prone individuals through the expression of cytokines by immune cells. In addition, similarities between self-antigens and the COVID-19 spike protein can lead to activation of immune pathways and targeting of self-antigens, thereby contributing to the development of myocarditis [21].

Thus, both ICIs and COVID-19-induced myocarditis are suspected to share a common mechanistic pathway of inappropriate immune overreaction leading to myocardial targeting and damage.

In our cohort, all types of myocarditis showed a male predominance, as seen in previous studies [22]. Because the ICIs population was significantly older, an expected higher prevalence of cardiac risk factors and chronic medical therapy was observed among that group. However, there was no significant difference in baseline cardiac diseases between the three groups. Previous studies have highlighted the differences in the presenting symptoms between ICI-induced myocarditis and viral myocarditis. Typically, ICI-induced myocarditis has presented with non-specific symptoms like fatigue and dyspnea, while viral myocarditis will present primarily with chest pain and palpitations [23]. A case series previously reported that all patients with COVID-19 vaccine-induced myocarditis presented with chest pain and most of them were found to have ST-elevations on ECG [7]. Similar to the previous studies mentioned, we observed that dyspnea was the main symptom of the ICI group, while chest pain and fever were the most common chief complaints in both the COVID-19 vaccine and Viral groups.

Inherent to the diagnosis of myocarditis, all patients were found to have elevated serum troponin levels. Interestingly, although the ICI group was older and sicker, the levels of hs-TnI were significantly lower (by 11–25-fold) than in the other two groups.

Past studies have shown that LV dysfunction, as manifested by reduced LVEF, is considered to be more common among patients diagnosed with viral myocarditis, and can be seen in up to 70% of this population [24]. Mahmood et al. [19] showed that only approximately 50% of patients diagnosed with ICIs-induced myocarditis presented with an LVEF or less than 50%. Upon evaluation of the echocardiography parameters in our cohort, we observed no significant differences in the LVEF between the groups, with an overall normal median value. However, significant differences were observed in 2D–STE parameters. These measurements are considered to be more sensitive to early minor myocardial damage and overall LVGLS was found to be significantly reduced in the ICI group compared to the other groups. In accordance with our results, Awadalla et al. [24] presented similar lower absolute LVGLS values (14.1%) among patients diagnosed with ICI-induced myocarditis. This may imply that ICI-induced myocarditis reactions cause early subclinical myocardial damage, and therefore need to be evaluated over a longer time period to monitor progression. Of significance, Awadalla et al. showed that reduced LVGLS is associated with the development of major cardiovascular adverse events (MACE) [24]. While the lower LVGLS might be the cause of the older age and medical history of the ICIs populations, when evaluating the LVGLS in follow-up echo, higher LVGLS values were observed (mean-16.2%) suggesting that the cause for LVGLS was the acute stage of myocarditis. Furthermore, diastolic dysfunction, estimated both by filling pressure and LAS, was more impaired in the ICI group when compared to the other two groups. Previous reports suggested that LAS is reduced in patients treated with anthracycline therapy [25]. However, data regarding LAS in patients with ICIs and COVID-19 vaccine-induced myocarditis is lacking. Our finding of reduced LAS values among the ICI group supports the theory of early subclinical damage, not observed yet by LVEF. According to past studies, these significant lower values cannot be explained solely by the older age of the ICI group population [26].

While suspected to share a common mechanism, our results reveal different clinical and imaging presentations between ICIs and COVID-19 vaccine-induced myocarditis. This might be explained by the difference in the targeting of each technology—ICIs targeting PD-L1 compared to the COVID-19 vaccine targeting the COVID-19 mRNA. Each target may have a different role and expression in the myocardium. Future studies evaluating the specific mechanism of each manifestation are needed.

The protocolized therapy for ICIs-induced myocarditis differs significantly from the therapy for COVID-19 vaccine and Viral groups. While the primary focus is directed on immunosuppression therapy in the ICI group, cardioprotective therapy is the focus in post-COVID-19 vaccine patients and anti-inflammatory therapy is the mainstay for viral myocarditis patients.

Our study has several limitations; first, it is a single-center retrospective study, and therefore our results are subject to the effects of possible confounders and may be biased by the nature of this design. Second, we recognize that the relatively small sample size reduces the statistical power of our results. Third, we realize that the significant differences in baseline characteristics, mainly age, could mislead the results, however, we believe that age and the history of chronic disease can’t explain the differences in symptoms, troponin level or the significant lower 2D–STE we observed. Last, the lack of endomyocardial biopsy limits the understanding of the difference in the mechanism of the groups; however, due to its invasive nature, the use of biopsy is globally limited and not part of the routine evaluation, and therefore the focus of advanced imaging, as 2D–STE is extremely important.

## 5. Conclusions

Our study found that, despite sharing similar probable pathogenic mechanisms, ICI and COVID-19 vaccine-induced myocarditis have significantly different clinical, laboratory, and imaging characteristics. Patients with COVID-19 vaccine-induced myocarditis have a more classical and benign presentation, similar to viral myocarditis, while patients with ICI-induced myocarditis presented with a non-classical presentation, including earlier myocardial damage as assessed by 2D–STE. To the best of our knowledge, this is the first study to assess LAS and RV strain in patients with ICI-induced myocarditis. Our findings suggest that routine 2D–STE surveillance may detect early cardiac damage and larger studies with longer follow-up are needed.

## Figures and Tables

**Table 1:** Baseline characteristics.

	ICI Group *n* = 9	COVID-19 Vaccinee Group *n* = 9	Viral Group *n* = 20	*p*-Value All
Age (years), median (IQR)	74 (64–79)	20(19–29)	24(22–26)	**<0.001**
Female gender, % (n)	22% (2)	11% (1)	10% (2)	0.815
Prior myocarditis, % (n)	0 (0)	11% (1)	10% (2)	>0.999
Hypothyroidism, % (n)	11% (1)	0 (0)	0 (0)	0.473
Ischemic Heart Disease, % (n)	11% (1)	0 (0)	0 (0)	0.473
Atrial Fibrillation, % (n)	22% (2)	0 (0)	0 (0)	0.096
Stroke, % (n)	0 (0)	0 (0)	0 (0)	n/a
Smoker, % (n)	33% (3)	11% (1)	33% (3)	0.613
Hyperlipidemia, % (n)	44% (4)	0 (0)	0 (0)	**0.004**
Hypertension, % (n)	33% (3)	0 (0)	0 (0)	**0.011**
Diabetes Mellitus, % (n)	11% (1)	0 (0)	0 (0)	0.244
**Baseline medications**				
ACEi/ARB, % (n)	33% (3)	0 (0)	0 (0)	**0.011**
Beta-blockers, % (n)	44% (4)	0 (0)	0 (0)	**0.003**
Statin, % (n)	22% (2)	0 (0)	0 (0)	0.096
Anticoagulation, % (n)	33% (3)	0 (0)	0 (0)	**0.021**
Anti-aggregation, % (n)	33% (3)	0 (0)	0 (0)	**0.021**

ACEi/ARB = angiotensin-converting enzyme inhibitor/angiotensin II receptor blocker, COVID-19 = coronavirus disease 2019, ICI = immune checkpoint inhibitors, IQR = interquartile range, *n* = number.

**Table 2:** Presentation of clinical tests.

	ICI Group *n* = 9	COVID-19 Vaccinee Group *n* = 9	Viral Group *n* = 20	*p*-Value All
**Symptoms**				
Chest pain, % (n)	44% (4)	100% (9)	90% (18)	**0.010**
Fever, % (n)	0 (0)	78% (7)	40% (8)	**0.002**
Dyspnea, % (n)	67% (6)	11% 1)	5% (1)	**0.001**
Pericardial rub, % (n)	0 (0)	0 (0)	10% (2)	>0.999
**Vital Signs**				
Heart Rate (beats per minutes), mean (SD)	90 (15)	80(13)	75(13)	0.054
Systolic Blood Pressure (mmHg), mean (SD)	127 (22)	121(21)	119(14)	0.551
Diastolic Blood Pressure (mmHg), mean (SD)	70 (15)	78(12)	73(12)	0.446
**Blood Tests**				
Troponin peak (ng/L), median (IQR)	619 (204–1542)	15,527 (5024–22,766)	7388 (1821–27,323)	**0.004**
CPK peak (U/L), median (IQR)	726 (37–7957)	698(458–2734)	189 (126–349)	0.054
CRP peak (mg/L), median (IQR)	61 (9–124)	44 (13–81)	39 (25–114)	0.899
Hemoglobin (g/dL), median (IQR)	10.6 (9.8–13.7)	15.2 (13.9–15.7)	13.8 (13.3–14.9)	**0.001**
WBC median (10^3/µL), median (IQR)	5.9 (4.5–9.8)	9.8 (7.4–11.9)	8.6 (6.9–11.6)	0.064
NLR, median (IQR)	5.8 (3–11.1)	3.8 (3.1–5.8)	2.6 (1.6–4.5)	0.266
PLT (10^3/µL), median (IQR)	190 (144–263)	189 (179–227)	221 (197–248)	0.282
Creatinine (mg/dL), median (IQR)	0.7 (0.6–1.3)	0.9 (0.8–1.0)	0.9 (0.7–1.1)	0.612
Triglycerides (mg/dL), median (IQR)	112 (76–169)	79 (57–89)	88 (73–112)	0.243
Cholesterol (mg/dL), median (IQR)	165 (138–221)	144 (130–179)	144 (115–161)	0.123
TSH (mu/L), median (IQR)	5.8 (1.4–29)	1.1 (1–1.4)	1.4 (0.8–2.2)	0.056
**Initiation of medical therapy**				
NSAIDS, % (n)	0 (0)	0 (0)	45% (9)	**0.007**
Aspirin, % (n)	22% (2)	56% (5)	45% (9)	0.386
Corticosteroids, % (n)	78% (7)	0 (0)	0 (0)	**<0.001**
Colchicine, % (n)	0 (0)	56% (5)	85% (17)	**<0.001**
ACEi/ARB, % (n)	33% (3)	89% (8)	60% (12)	0.054
Beta-blockers, % (n)	67% (6)	89% (8)	55% (11)	0.209

COVID-19 = coronavirus disease 2019, n = number, CPK = creatinine phospho–kinase, CRP = c-reactive protein, ICI = Immune checkpoint inhibitors, IQR = interquartile range NLR = neutrophil-lymphocyte ration, PLT = platelets, TSH = thyroid stimulating hormone, WBC = white blood cells, NSAIDS = non-steroidal anti-inflammatory drugs, ACEi/ARB = angiotensin-converting enzyme inhibitor/angiotensin II receptor blocker.

**Table 3:** Echocardiographic and electrocardiographic parameters.

	ICI Group *n* = 9	COVID-19 Vaccine Group *n* = 9	Viral Group *n* = 20	*p*-Value All
**Electrocardiography**				
PR depression, % (n)	11% (1)	11% (1)	10% (2)	>0.999
ST elevation, % (n)	0 (0)	67% (6)	60% (12)	**0.004**
Inverted T wave, % (n)	33% (3)	22% (2)	30% (6)	>0.999
**Echocardiography parameters**				
Pericardial effusion, % (n)	0 (0)	0 (0)	20% (4)	0.151
Ejection Fraction (%), median (IQR)	60 (40–60)	60 (50–60)	60 (56–60)	0.385
LVEDd (mm), mean (SD)	48 (±10)	48 (±2)	50 (±3)	0.427
LVESd (mm), mean (SD)	32 (±10)	31 (±5)	34 (±3)	0.364
IVSd (mm), mean (SD)	11 (8–12)	8 (7–10)	8 (8–9)	**0.021**
E/A, mean (SD)	0.8 (±0.3)	1.8 (±0.7)	1.7 (±0.5)	**0.002**
Deceleration Time (milliseconds), mean (SD)	178 (±26)	182 (±40)	171 (±46)	0.766
e’ septal, mean (SD)	7 (±2)	10 (±2)	12 (±2)	**<0.001**
e’ lateral, mean (SD)	9 (±1)	14 (±4)	15 (±3)	**<0.001**
E/e’ septal, mean (SD)	10 (±3)	8 (±2)	7 (±2)	**0.010**
E/e’ lateral, mean (SD)	8 (±3)	6 (±2)	5 (±1)	**0.007**
E/e’ average, mean (SD)	10 (±4)	7 (±2)	6 (±1)	**0.002**
LAVI (mL/m^2^), mean (SD)	31 (±10)	26 (±5)	30 (±7)	0.389
TAPSE (mm), mean (SD)	22 (±4)	23 (±5)	23 (±3)	0.926
SPAP (mmHg), mean (SD)	30 (±12)	25 (±7)	24 (±4)	0.367
**Speckle Strain**				
LV GLS (%), mean (SD)	13 (±5)	17 (±2)	18 (±4)	**0.016**
RVFWS (%), mean (SD)	24 (±1)	21 (±4)	24 (±4)	0.243
RV4CSL (%), mean (SD)	17 (±4)	18 (±3)	20 (±4)	0.252
LA reservoir (%), mean (SD)	32 (±12)	33 (±12)	44 (±14)	0.066
LA conduit (%), mean (SD)	17 (±11)	30 (±7)	37 (±11)	**0.001**
LA pump (%), mean (SD)	19 (±8)	7 (±3)	10 (±4)	**0.001**

COVID-19 = coronavirus disease 2019, ICI = immune checkpoint inhibitors, IQR = interquartile range, IVS = interventricular septum, LA = left atrium, LAVI = left atrium volume index, LVEDd = left ventricular end diastolic dimension, LVESd = left ventricular end systolic dimension, LV GLS = left ventricle global longitudinal strain, n = number, RV4CSL = global 4-chamber contour, RVFWLS = free wall longitudinal strain, SPAP = systolic pulmonary atrial pressure, mmHg, TAPSE = tricuspid annular plane systolic excursion.

## Data Availability

Not applicable.
